# Frequent Novel Viral Focal Copy Number Gains Potentiate Radiosensitivity in Natural Killer/T-Cell Lymphoma

**DOI:** 10.34133/cancomm.0055

**Published:** 2026-09-30

**Authors:** Jing Quan Lim, The Phyu, Dachuan Huang, Beng Hooi Phang, Ziyu Fang, Kelila Chai Xin Ye, Nur Ayuni Binte Muhammad Taib, Wei Yi Ng, June Xin Ni Wong, Wendy Lee, Kerry May Lim Huifen, Liang Wei Wang, Rou-Jun Peng, Yi Xia, Yi-Qi Li, Anand Jeyasekharan, Soo Yong Tan, Chee Leong Cheng, Nagavalli Somasundaram, Shin Yeu Ong, Wee-Joo Chng, Bernett Lee, Sai Mun Leong, Olaf Rötzschke, Lee-Yung Shih, Jing Tan, Jason Yongsheng Chan, Jie Xiong, Wei-Li Zhao, Fei Yao, Jianjun Liu, Soon Thye Lim, Jin-Xin Bei, Chit Fang Cheok, Siok-Bian Ng, Choon Kiat Ong

**Affiliations:** ^1^ Lymphoma Translational Research Laboratory, Cellular and Molecular Research, National Cancer Centre Singapore, Singapore, Singapore.; ^2^ Oncology Academic Clinical Programme, Duke-NUS Medical School, Singapore, Singapore.; ^3^ Department of Pathology, Yong Loo Lin School of Medicine, National University of Singapore, Singapore, Singapore.; ^4^ Singapore Immunology Network, Agency for Science, Technology and Research, Singapore, Singapore.; ^5^ Department of Medical Oncology, State Key Laboratory of Oncology in South China, Guangdong Provincial Clinical Research Center for Cancer, Sun Yat-sen University Cancer Center, Guangzhou, Guangdong, P. R. China.; ^6^ Department of Haematology-Oncology, National University Health System, Singapore, Singapore.; ^7^ Cancer Science Institute of Singapore, National University of Singapore, Singapore, Singapore.; ^8^ NUS Centre for Cancer Research, Yong Loo Lin School of Medicine, National University of Singapore, Singapore, Singapore.; ^9^ Department of Pathology, Singapore General Hospital, Singapore, Singapore.; ^10^ Division of Medical Oncology, National Cancer Centre Singapore, Singapore, Singapore.; ^11^ Department of Haematology-Oncology, National University Cancer Institute, National University Health System, Singapore, Singapore.; ^12^ Centre for Biomedical Informatics, Lee Kong Chian School of Medicine, Nanyang Technological University, Singapore, Singapore.; ^13^ Division of Hematology-Oncology, Chang Gung Memorial Hospital at Linkou, Taoyuan, Taiwan, China.; ^14^ Chang Gung University, Taoyuan, Taiwan, China.; ^15^ Laboratory of Cancer Epigenome, Division of Medical Sciences, National Cancer Centre Singapore, Singapore, Singapore.; ^16^ Cancer Discovery Hub, National Cancer Centre Singapore, Singapore, Singapore.; ^17^ Shanghai Institute of Hematology, State Key Laboratory of Medical Genomics, National Research Center for Translational Medicine at Shanghai Ruijin Hospital, Shanghai Jiao Tong University School of Medicine, Shanghai, P. R. China.; ^18^ Genome Institute of Singapore, Agency for Science, Technology and Research, Singapore, Singapore.; ^19^ Director’s Office, National Cancer Centre Singapore, Singapore, Singapore.; ^20^ Office of Education, Duke-NUS Medical School, Singapore, Singapore.; ^21^ Institute of Molecular and Cellular Biology, Agency for Science, Technology and Research, Singapore, Singapore.; ^22^ Cancer and Stem Cell Biology Program, Duke-NUS Medical School, Singapore, Singapore.; ^23^ Center of Excellence for Translational Medicine, Fundeni Clinical Institute, Bucharest, Romania.

Epstein–Barr virus (EBV)-associated natural killer/T-cell lymphoma (NKTCL) is an aggressive malignancy prevalent in Asia, for which radiotherapy remains central to treatment, particularly in limited-stage disease [1]. Although recurrent host-genome alterations are well characterized [2,3], structural variants have been associated with treatment response [4] and the clinical relevance of structural variation within EBV remains unclear [5,6]. Whether viral genomic alterations contribute to treatment response has not been systematically examined [7].

Whole-genome sequencing (WGS) data of 77 tumor–normal paired NKTCL samples (mean depths: 68.5× tumor and 39.9× matched normal; Table S1) identified short variants enriched at 85 to 86 and 168 to 169 kbp (Fig. 1A, 99th percentile among 1,000-bp bins). Consistent with a recent study [6], the dominant hotspot (168 to 169 kbp) overlapped with the 5′ end of the latent membrane protein 1 (*LMP-1*) gene, a major EBV latent membrane protein. The second hotspot, within *LMP-1*, was a 15-bp (p.276_281del) in-frame deletion within the C-terminal activating region 3 (CTAR3) domain (Fig. S1), which has been reported to bind to Janus kinase 3 (JAK3) and activate signal transducer and activator of transcription (STAT) proteins that were commonly found with activating somatic mutations [8,9].

**Fig. 1.**
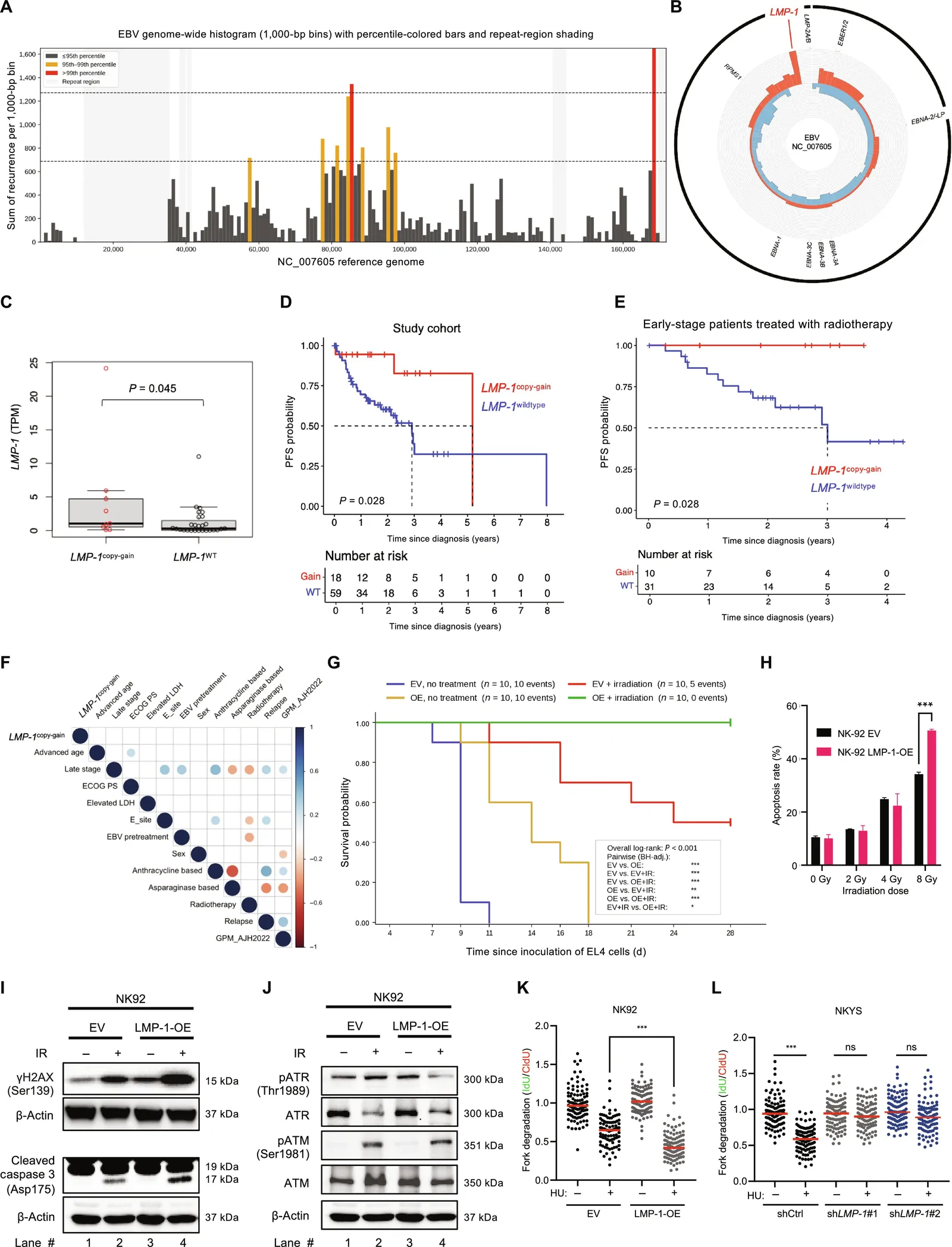
The genomic copy number gain of viral *LMP-1* locus induces replication fork stalls for sensitivity to radiotherapy in natural killer/T-cell lymphoma. (A) Manhattan histogram plot of 1,000-bp bins on nonsilent short-variant mutations across the whole of the NC_007605 EBV reference genome. The top 99th and 95th percentile bins are colored in red and orange, respectively, and the remaining bins are in dark gray. Large repeats, more than 1,000 bp in size, are highlighted in light gray. (B) CIRCOS histograms plot of the copy-gain (red) and copy-loss (blue) events over the reference NC_007605 of 77 EBV genomes in NKTCL. (C) The TPM expression counts of the viral *LMP-1*, as profiled by RNA sequencing, in tumors harboring *LMP-1*^copy-gain^ and *LMP-1*^WT^. (D) The KM curves depicting the progression-free survival of patients with NKTCL harboring *LMP-1*^copy-gain^ or *LMP-1*^WT^ tumors. (E) KM curves depicting the progression-free survival of patients with early-stage NKTCL, who received radiotherapy, harboring *LMP-1*^copy-gain^ or *LMP-1*^WT^ tumors. (F) Pearson correlogram between the clinical risk features, molecular markers, treatment modalities, and genomic prognostic markers of the study cohort. GPM_AJH2022 is the genomic prognostic model for NKTCL. Circle color represents the direction of correlation, whereas circle size and color intensity represent magnitude. Blue indicates positive correlations, and red indicates negative correlations. Larger, darker circles denote stronger correlations, with coefficients approaching +1 or −1; smaller, paler circles indicate weaker correlations approaching 0. Dark-blue diagonal circles represent self-correlations (coefficient = 1). Only one-half of the symmetric correlation matrix is displayed. Blank cells indicate correlations that did not meet the specified significance threshold (*P* ≥ 0.05). (G) KM survival of EL4 (EV or LMP-1-OE) tumor-bearing C57BL/6J mice ± local irradiation (4 Gy on days 8 and 15). *n* = 10/arm with 6 males and 4 females per arm, with events recorded as death, humane endpoint met, or tumor volume exceeding 2,000 mm^3^. (H) Quantification of apoptotic cells in NK92, comparing the effects of *LMP-1* overexpression (LMP-1-OE) and control EV, after exposure to varying doses of irradiation. (I) Western blot analysis of γH2AX (for DNA damage) in both control and LMP-1-OE NK92 cells, with and without exposure to 8 Gy of irradiation, post-8 h. Additionally, western blots of cleaved caspase 3 (for apoptotic activity) and (J) ATR/ATM (for DNA damage response) were profiled at 24 h postirradiation. (K) Quantitative assessment of fork degradation (IdU-/CldU-tract length ratios) for control EV and LMP-1-OE on either untreated or treated with HU (2 mol·m^−3^) is presented in a scatterplot (*n* >100 fibers). (L) Quantitative assessments of fork degradation (IdU-/CldU-tract length ratios) for shCtrl, sh*LMP-1*#1 and sh*LMP-1*#2 on either untreated or treated with HU (5 mol·m^−3^) are presented in a scatterplot (*n* > 100 fibers). Red bars represent the mean IdU-/CldU-tract length ratios. Statistical significance was determined using ANOVA for apoptosis assay and comparison of tumor volumes, Mann–Whitney 2-sided test for DNA fiber assay, and log-rank test for KM curves: *P < 0.05, **P < 0.01, ***P < 0.001. Abbreviations: EBV, Epstein–Barr virus; bp, base pair; TPM, transcripts per million; *LMP-1/2A/2B*, latent membrane protein 1/2A/2B; *EBER1/2*, Epstein–Barr virus-encoded RNA 1/2; *EBNA1/2/3A/3B/3C-LP*, Epstein–Barr virus nuclear antigen 1/2/3A/3B/3C/-leader protein; *RPMS1*, rightward reading frame in the BamHI-M and BamHI-S regions 1; gain, *LMP-1*^copy-gain^; WT, wild-type; PFS, progression-free survival; ECOG PS, Eastern Cooperative Oncology Group performance status; LDH, lactate dehydrogenase; E_site, extranodal site more than 1; GPM, genomic prognostic model; IR, irradiation; NKTCL, natural killer/T-cell lymphoma; KM, Kaplan–Meier; BH, Benjamini–Hochberg correction; adj, adjusted; EV, empty vector; OE, overexpression; IdU, 5-iodo-2′-deoxyuridine; CldU, 5-chloro-2′-deoxyuridine; HU, hydroxyurea; ANOVA, analysis of variance; ns, not significant; γH2AX, gamma H2A histone family member X; β-actin, beta-actin; pATR, phosphorylated ataxia–telangiectasia and Rad3-related; pATM, phosphorylated ataxia–telangiectasia mutated protein; kDa, kilodaltons.

Analysis of the EBV CNV landscape identified losses at the rightward reading frame in the BamHI-M and BamHI-S regions 1 (*RPMS1*) locus in 11.7% (9/77) of tumors, as reported in chronic active EBV infection and EBV-positive diffuse large B-cell lymphoma (Fig. S2 and Table S2) [5]. Notably, gains at the *LMP-1* locus (*LMP-1*^copy-gain^) occurred in 23.4% (18/77; Fig. 1B) of tumors and 11.1% (1/9) of NKTCL cell lines. Breakpoints were independently validated in viral isolates from 2 tumors (Fig. S3) and the NKYS cell line (Fig. S4) using short- and long-read resequencing (Fig. S5). Across an additional 703 EBV-positive genome-wide sequencing datasets, *LMP-1*^copy-gain^ was observed in EBV-associated hematolymphoid malignancies (*P* < 0.001; Table S3), predominantly NKTCL, suggesting that it is a prominent NKTCL feature.

RNA sequencing showed 3.68× higher *LMP-1* levels (*P* = 0.045) in *LMP-1*^copy-gain^ than *LMP-1*^WT^ tumors (Fig. 1C). However, EBV viral loads correlated weakly with *LMP-1* expression but strongly with relative *LMP-1* copy number (Pearson rho = 0.77, *P* < 0.001; Table S4), suggesting that copy-gain led to increased expression of *LMP-1*.

Interestingly, a meta-analysis associated *LMP-1* expression with worse survival across EBV-associated malignancies (hazard ratio [HR] = 1.51, 95% confidence interval [CI] 1.13 to 2.03; *P* < 0.001) [10]. However, *LMP-1*^copy-gain^ tumors in our cohort trended with longer progression-free survival (PFS: HR = 0.28, 95% CI 0.08 to 0.94; *P* = 0.028, Fig. 1D) and overall survival (HR = 0.27, 95% CI 0.06 to 1.16; *P* = 0.060, Fig. S6). This association was further supported in the subgroup of early-stage patients who received radiotherapy, in which no PFS events occurred among patients with *LMP-1*^copy-gain^, resulting in significantly longer PFS compared with *LMP-1*^WT^ patients (*P* = 0.028, Fig. 1E), with a similar trend observed for overall survival (*P* = 0.086, Fig. S7). *LMP-1*^copy-gain^ was unrelated to baseline clinical risk features (Fig. 1F) and remained independently associated with improved PFS after clinical risk adjustment (Table S5). However, our retrospective heterogeneous cohort can neither distinguish whether *LMP-1*^copy-gain^ is prognostic or predictive of radiosensitivity nor exclude residual confounding. Nevertheless, we hypothesized that *LMP-1* overexpression (LMP-1-OE) could sensitize NKTCL to radiotherapy.

To test this hypothesis in vivo, we implanted EL4 murine lymphoma cells transfected with LMP-1-OE vector or empty vector (EV) (Fig. S8). Irradiation improved survival versus nonirradiated controls in both groups (*P* < 0.001; Fig. 1G and Fig. S9), but irradiated EV tumors resumed growth after a transient delay, whereas irradiated LMP-1-OE tumors showed sustained regression (Fig. S10). These findings support enhanced radiosensitivity in vivo. Next, to study the functional consequences of *LMP-1*^copy-gain^, NK92 (*LMP-1*^low^) and NKYS (*LMP-1*^high^) were selected for LMP-1-OE and knockdown (*LMP-1*^KD^) experiments, respectively (Fig. S11). Upon irradiation, both control and LMP-1-OE NK92 cells exhibited a radiation-dose-dependent increase in apoptosis, with LMP-1-OE NK92 cells having significantly higher irradiation-induced apoptosis (*P* < 0.001) at 48 h post-8-Gy treatment (Fig. 1H and Fig. S12). Conversely, NKYS *LMP-1*^KD^ had consistently lower irradiation-induced apoptosis than shCtrl cells (Fig. S12). Cleaved caspase 3 increased in irradiated LMP-1-OE NK92 cells (Fig. 1I, lane 2 vs. lane 4) and decreased in irradiated *LMP-1*^KD^ NKYS cells (Fig. S13, lane 2 vs. lanes 4 and 6). LMP-1 overexpression increased gamma H2A histone family member X (γH2AX) before irradiation (Fig. 1I, lane 1 vs. lane 3), with a larger increase after irradiation (Fig. 1I, lane 2 vs. lane 4), indicating enhanced DNA damage. Along with increased γH2AX and apoptosis, we also observed that phosphorylated ataxia–telangiectasia and Rad3-related (pATR) was increased in control EV cells upon irradiation (Fig. 1J, lane 1 vs. lane 2) but was markedly decreased in the LMP-1-OE cells (Fig. 1J, lane 3 vs. lane 4). In contrast, phosphorylated ataxia–telangiectasia mutated protein (pATM) remained unchanged between control EV cells and LMP-1-OE cells after irradiation (Fig. 1J, lane 2 vs. lane 4). Together with increased γH2AX and cleaved caspase 3, these findings are consistent with altered ATR-mediated replication stress responses in LMP-1-OE cells and are compatible with increased genomic instability after irradiation.

As LMP-1-OE sensitized NKTCL cells to radiation through the ATR replication stress pathway, we examined replication fork integrity with DNA fiber assay (Fig. S14). Cells were labeled with halogenated nucleotides, 5-chloro-2′-deoxyuridine (CldU) and 5-iodo-2′-deoxyuridine (IdU), before hydroxyurea (HU)-induced fork arrest. HU reduced IdU/CldU ratios in EV NK92 cells, indicating nascent-DNA degradation (Fig. 1K). Ratios were further reduced in LMP-1-OE cells, indicating that LMP-1-OE promotes replication stress through impaired fork stability.

To confirm the role of LMP-1 in compromising fork integrity, in part by promoting fork degradation, we performed LMP-1 knockdown in NKYS cells, which exhibit high LMP-1 expression. Consistently, pronounced fork degradation was observed in NKYS cells treated with HU (Fig. 1L). Knockdown of LMP-1 using 2 different short hairpin RNAs restored fork protection, rendering cells resistant to fork degradation under replication stress. Altogether, our results corroborate the finding that LMP-1 plays a previously undescribed role in replication stress by promoting fork degradation. Consistent with the idea that LMP-1 overexpression increases DNA damage in response to irradiation, potentially by altering DNA damage responses, we demonstrated that LMP-1 overexpression exacerbated replication stress by promoting fork degradation. The role of *LMP-1* in replication fork dynamics warrants further investigation.

*LMP-1* expression is a defining feature of EBV latency II and III infection programs. As the EBV in NKTCL predominantly exhibits latency II infection, the biological consequences of *LMP-1*^copy-gain^ may be particularly relevant within this latency context. Whether they preferentially occur within specific EBV latency states remains an important question for future investigation. Additionally, *LMP-1*-driven replication stress could also be influenced by the mutational landscape of the host genome, as frequent mutations of genes involved in preserving genome stability, JAK–STAT signaling, and other cell-cycle regulatory pathways have also been found in NKTCL [2]. Lastly, several subgroup analyses, particularly those restricted to radiotherapy-treated early-stage disease, were performed on relatively small patient numbers. In view of these limitations, larger prospective cohorts will be required to elucidate potential host–viral mutational interactions in *LMP-1*-driven replication stress and validate our findings.

In conclusion, focal *LMP-1*^copy-gain^ was associated with increased LMP-1 expression and favorable outcomes after radiotherapy in NKTCL. Functional studies suggest that LMP-1 overexpression enhances radiosensitivity by promoting replication stress and impairing fork protection. Across EBV-associated malignancies, the effects of *LMP-1*^copy-gain^ may depend on disease and treatment context, highlighting how host–viral genome interactions may influence clinical outcomes.

## Ethical Approval

This study was approved by the Institutional Review Board of SingHealth (2004/407/F), a domain-specific review board from the National Healthcare Group (DSRB2015/00176), and the review board of Sun Yat-sen University Cancer Center (YB2015-015-01). Either a waiver of consent or written informed consents were obtained for all patients in this study. All mouse experiments were approved by the SingHealth Institutional Animal Care and Use Committee (2023/SHS/1818).

## Data Availability

The data sources of all analyzed samples are detailed in Table S6. The in-house generated WGS data of 50 tumor–normal paired NKTCL samples were deposited in the European Genome-phenome Archive (EGA) with accession number EGAD00001005231. The WTS data of 36 NKTCL tumors were deposited into EGA with accession number EGAD00001005230. The Nanopore WGS long-read sequencing data of the NKYS cell line were deposited into EGA with accession number EGAD50000000381. Three NKTCLs were also resequenced by both Illumina short-read and Nanopore long-read sequencing, and their data have been deposited at the National Center for Biotechnology Information’s (NCBI’s) Sequence Read Archive (SRA) with Project ID PRJNA1473513. A public WGS dataset of 295 EBV isolates from 18 Burkitt lymphomas (BLs), 126 chronic active EBV infections, 35 diffuse large B-cell lymphomas, 27 NKTCLs, 29 Hodgkins lymphomas (HLs), 10 hemophagocytic lymphohistiocytosis, 13 infectious mononucleosis, 29 post-transplant lymphoproliferative disorders, and 8 other unspecified cases was downloaded from the NCBI database SRA with Project ID DRP013409. Another public WGS dataset of EBV isolates containing 2 BLs, 16 gastric carcinomas, 54 healthy controls, 11 HLs, 7 non-Hodgkins lymphomas, and 180 nasopharyngeal carcinomas (NPCs) was downloaded from the NCBI database (BioProject ID: PRJNA522388). Another public WGS dataset of 41 EBV isolates from endemic BLs was downloaded from the SRA repository (accession number: SRP212943). Three public WGS datasets of 27 NKTCLs, 12 NPCs, and 15 NPCs were downloaded from the National Genomics Data Center (Experiment ID: OEX001947), SRA repository (accession number: SRP126796), and European Nucleotide Archive (ENA) (accession number PRJEB12830), respectively.
