# Supplementary material for: Frequent Novel Viral Focal Copy Number Gains Potentiate Radiosensitivity in Natural Killer/T-Cell Lymphoma
Source: Cancer Commun (Lond). 2026 Sep 30;46:0055. doi: 10.34133/cancomm.0055 (PMC13624365; doi:10.34133/cancomm.0055)
Supplement: Supplementary file 1 [file cancomm.0055.f1.zip › CANCOMM-D-26-00332-Supplementary-Final.docx]

**Supplementary Materials for**

**Frequent Novel Viral Focal Copy-Gains Potentiate Radiosensitivity in Natural Killer/T-cell Lymphoma**

Jing Quan Lim^1,2†*^, The Phyu^3†^, Dachuan Huang^1,2†^, Beng Hooi Phang^1^, Ziyu Fang^3^, Kelila Chai Xin Ye^1^, Nur Ayuni Binte Muhammad Taib^1^, Wei Yi Ng^1^, June Xin Ni Wong^3^, Wendy Lee^4^, Kerry May Lim Huifen^1^, Liang Wei Wang^4^, Rou-Jun Peng^5^, Yi Xia^5^, Yi-Qi Li^5^, Anand Jeyasekharan^6,7^, Soo Yong Tan^3,8^, Chee Leong Cheng^9^, Nagavalli Somasundaram^10^, Shin Yeu Ong^9^, Wee-Joo Chng^7,8,11^, Bernett Lee^12^, Sai Mun Leong^3,8^, Olaf Rötzschke^4^, Lee-Yung Shih^13,14^, Jing Tan^5,15^, Jason Yongsheng Chan^11,16^, Jie Xiong^17^, Wei-Li Zhao^17^, Fei Yao^18^, Jianjun Liu^18^, Soon Thye Lim^19,20^, Jin-Xin Bei^5^, Chit Fang Cheok^3,8,21^, Siok-Bian Ng^3,7,8*^, Choon Kiat Ong^1,22,23*^

^1^Lymphoma Translational Research Laboratory, Cellular and Molecular Research, National Cancer Centre Singapore, Singapore, Singapore.

^2^Oncology Academic Clinical Programme, Duke-NUS Medical School, Singapore, Singapore.

^3^Department of Pathology, Yong Loo Lin School of Medicine, National University of Singapore, Singapore, Singapore.

^4^Singapore Immunology Network, Agency for Science, Technology and Research, Singapore, Singapore.

^5^Department of Medical Oncology, State Key Laboratory of Oncology in South China, Guangdong Provincial Clinical Research Center for Cancer, Sun Yat-sen University Cancer Center, Guangzhou, Guangdong, P. R. China.

^6^Department of Haematology-Oncology, National University Health System, Singapore, Singapore.

^7^Cancer Science Institute of Singapore, National University of Singapore, Singapore, Singapore.

^8^NUS Centre for Cancer Research, Yong Loo Lin School of Medicine, National University of Singapore, Singapore, Singapore.

^9^Department of Pathology, Singapore General Hospital, Singapore, Singapore.

^10^Division of Medical Oncology, National Cancer Centre Singapore, Singapore, Singapore.

^11^Department of Haematology-Oncology, National University Cancer Institute, National University Health System, Singapore, Singapore.

^12^Centre for Biomedical Informatics, Lee Kong Chian School of Medicine, Nanyang Technological University, Singapore, Singapore.

^13^Division of Hematology-Oncology, Chang Gung Memorial Hospital at Linkou, Taoyuan, Taiwan, China.

^14^Chang Gung University, Taoyuan, Taiwan, China.

^15^Laboratory of Cancer Epigenome, Division of Medical Sciences, National Cancer Centre Singapore, Singapore, Singapore.

^16^Cancer Discovery Hub, National Cancer Centre Singapore, Singapore, Singapore.

^17^Shanghai Institute of Hematology, State Key Laboratory of Medical Genomics, National Research Center for Translational Medicine at Shanghai Ruijin Hospital, Shanghai Jiao Tong University School of Medicine, Shanghai, P. R. China.

^18^Genome Institute of Singapore, Agency for Science, Technology and Research, Singapore, Singapore.

^19^Director’s Office, National Cancer Centre Singapore, Singapore, Singapore.

^20^Office of Education, Duke-NUS Medical School, Singapore, Singapore.

^21^Institute of Molecular and Cellular Biology, Agency for Science, Technology and Research, Singapore, Singapore.

^22^Cancer and Stem Cell Biology Program, Duke-NUS Medical School, Singapore, Singapore.

^23^Center of Excellence for Translational Medicine, Fundeni Clinical Institute, Bucharest, Romania.

^*^Address correspondence to:

J.Q.L. (Jing Quan Lim), lim.jing.quan@nccs.com.sg

C.K.O. (Choon Kiat Ong), cmrock@nccs.com.sg

S.B.N. (Siok-Bian Ng), patnsb@nus.edu.sg

^†^These authors contributed equally to this work

# Supplementary Materials and Methods

## Sample Cohort

A total of 50 nasal-type natural-killer/T cell lymphoma (NKTCL) cases were initially diagnosed according to the 2008 World Health Organization classification with cytotoxic, cluster of differentiation 3 epsilon positive (CD3ε+), and Epstein-Barr virus-encoded small RNA positive (EBER+) phenotypes [1]. An additional 27 NKTCLs with whole-genome sequencing (WGS) data were downloaded from a public repository (see the Data Availability section). All 77 NKTCL were nasal-type and of extranodal subtype, and could be reclassified as extranodal natural-killer/T cell lymphoma (ENKTL) under 2022 World Health Organization classification [2]. All NKTCLs profiled by WGS have matched-normal from either buccal swabs or blood, and 36 of the tumoral tissues had available total RNA for whole-transcriptome sequencing (WTS). The time-to-event survival data are shown in **Supplementary Table S7**. This study was approved by the Institutional Review Board of SingHealth (2004/407/F), a domain-specific review board from the National Healthcare Group (DSRB2015/00176), and the review board of Sun Yat-Sen University Cancer Center (YB2015-015-01). Detailed sample-level metadata is included as **Supplementary Table S7**. All the patients provided written informed consent in accordance with the Declaration of Helsinki.

## Whole-genome sequencing

Genomic DNA from 50 NKTCL tumors was extracted with the QIAamp DNA Mini Kit (Cat. #51304 and #51306, Qiagen). The DNA for the matching normal was obtained from blood or buccal swabs and purified with the Blood and Cell Culture DNA Mini kit (Cat. #13323, Qiagen) or E.Z.N.A. Tissue DNA Kit (Cat. #D3396, Omega Bio-tek), according to the manufacturer’s instructions. The quantity and quality were assessed using the Quant-iT PicoGreen dsDNA Assay Kit (Cat. #Q34250, Invitrogen) and agarose gel electrophoresis. All sequencing libraries were prepared with the TruSeq Nano DNA Library Prep Kit (Cat. #20015964, Illumina). Paired-end sequencing was performed on Illumina HiSeq 2000 or HiSeq X Ten for 2 × 101 bp and 2 × 151 bp reads, respectively. As a proxy for tumoral purity, each tumoral sample has upwards of 5000 Epstein-Barr virus (EBV)-aligned read-pairs.

## Nanopore long-read sequencing

The tumoral samples with at least 1 μg of leftover genomic DNA after WGS were subjected to targeted sequencing. The probe-set was designed by TWIST Biosciences and was based on the NC_007605 reference genome. EBV quantitative polymerase chain reaction was employed as a quality-control step to sequence samples with more than 500,000 viral copies per 500 ng DNA for nanopore long-read sequencing on the Promethion 2 R14 model M flow cell. Three patients samples, namely, NKTL6, NKTL7 and NKTL48, which already had WGS data, were resequenced as validatory samples.

## Whole-transcriptome sequencing

RNA extraction, and quality and quantity assessments were performed as previously described [3]. Sequencing libraries were prepared using the TruSeq Stranded Total RNA Library Prep Kit with Ribo-Zero (Cat. #RS-122-2201, Illumina), and WTS was performed on Illumina HiSeq 2500 and HiSeq 3000 for 2 × 101 bp and 2 × 151 bp reads, respectively.

## Pre-processing of DNA sequencing read

DNA sequencing adaptors, trailing low-quality bases and polyG sequences were removed by fastp (v0.19.6 [4]). The pre-processed reads were aligned using BWA-MEM (v0.7.17 [5]) to the hs37d5 human reference genome. Polymerase chain reaction (PCR) duplicates were subsequently marked by Sambamba (v0.6.5 [6]). The alignment coverage statistics were computed by Qualimap (v2.2.1 [7]).

## Mutational analysis of DNA sequencing reads

Somatic short-variants were called using Strelka2 (v2.9.4 [8]). Short-variant of the EBV genomes were called using Freebayes (v1.2.0 [9]). Meanwhile, wAnnovar (October 2021 [10]) was used to annotate the short-variants. The frequencies of non-silent viral mutations across our study cohort of 77 NKTCLs are detailed in **Supplementary Table S8**. The filtering criteria on the short-variants were further filtered against low complexity regions, dbSNP (v132 [11]) positions without corresponding annovar-annotated COSMIC IDs [12], simple repeats and homopolymers. CNV was called using CNVkit (v0.9.6 [13]) and GISTIC (v2.0.23 [14]) to identify significant CNVs. The candidate copy-change regions were merged into larger segments via the CNVkit’s “export seg” routine. Log2 thresholds of 0.4 and -0.6 were used to denote copy-gain and copy-loss for the WGS data, respectively. SRs were called using Manta (v1.6.0 [15]) and annotated by annotSV (v1.2 [16]). Each candidate SR was subjected to the following filtering criteria: 1) SR is supported by at least 3 discordant read-pairs and at least 3 soft-clipped reads, and the sum of all supporting reads is at least 10; 2) at least 10x coverage in both tumor and matching-normal data; and 3) SR is at least 1000 bp in size. Mutational driver-gene analysis was performed by ActiveDriverWGS (v1.1.1 [17]).

## Copy-number analysis of the EBV viral genomes

Annotated repetitive regions from National Center for Biotechnology Information Reference Sequence (NCBI RefSeq) (13 Aug 2018 [18]) were censored during copy-number variants (CNV) calling. Four samples, NKTL2, NKTL4, NKTL8, and NKTL9, with copy-neutral EBV genomes with >95% of its viral genomes being copy-neutral to the ‘anchor’ genomic window of BamHI A leftward reading frame 5 (*BALF5*) locus were being pooled together to form a single binary alignment/map file to form a pool-of-normals strategy for CNVkit, with additional parameters “--drop-low-coverage --target-avg-size=500”, to call single-sample/unmatched samples’ CNVs accurately (**Supplementary Figure S15**). The EBV load per tumoral cell was calculated by dividing the coverage of the NC_007605:55000-58000 locus by half of the coverage from the human tumoral genome (**Supplementary Table S4**). Log2 thresholds of 1 and -1 were used to denote preliminary copy-gain and copy-loss 500bp bins. Resultant copy-gain and copy-loss regions were supported by at least two adjacent non copy-neutral 500bp bins. We merged the corresponding copy-gain and copy-loss regions with bedtools (v2.25.0 [19]), with additional parameter “merge -d 500”. The genomic breakpoints of the viral copy-gains were checked for consistent aligned read-pairs orientations and also be supported by at least 10 clipped read-alignments.

Additional PCR-based Sanger sequencing were done on samples with available genomic materials. The primer-designs used for validating the viral copy-gains in NKTL6, NKTL7 and the human natural-killer NKYS cell line are as follow: NKTL6_lmp1_forward - 5’ CGTTGGAGGGTAGAATGACAGG 3’ and NKTL6_lmp1_reverse - 5’ CCTACATAAGCCTCTCTCACTGCT 3’ (size: 283 bp), NKTL7 _lmp1_forward - 5’ AGAGAGACCCTGGAATGTGTGT 3’ and NKTL7 _lmp1_reverse - 5’ GCGACCACATATGAGATTGAGA 3’ (size: 233 bp), and NKYS_lmp1_forward - 5’ CGCTCCTCACTTTCCAGTGT 3’ and NKYS_lmp1_reverse - 5’ CTGCAGATATCCTGCGTCCT 3’ (size: 230 bp).

## Quantification and normalization of RNA transcripts

RNA sequencing reads were aligned using STAR (v2.7.5a [20]) to a combined reference of hg19 and the EBV genome (NC_007605) in 2-pass mode 37. Gene quantification was performed using RSEM (v1.3.2 [21]). Gene counts were subsequently filtered against a blacklist containing ribosomal RNA, small nucleolar RNA, mitochondria-encoded, Metazoa signal recognition particle, and RN7 class of genes [18]. This was necessary to correct for the differences in the efficiency of the Ribo-Zero kits (Illumina) across multiple batches of library preparation. Gene counts were normalized to transcripts per million (TPM).

## Retroviral vector for the overexpression of LMP-1 in NK92 and EL4 cell lines

Packaging of retroviral particles was performed by co-transfecting the transfer vector pBABE-puro (#1764, Addgene) or MSCV-N-LMP1 (Cat. #37962, Addgene) with p-CMV-VSV-G (Cat. #8545, Addgene) and pCL-ECO (Cat. #12371, Addgene) into human embryonic kidney with SV40 T-antigen 293T cell line in antibiotic-free medium and incubated overnight. The medium was then refreshed with 15 mM HEPES buffered complete culture medium. At 48 h post-infection, the viral supernatant was centrifuged and syringe-filtered before introduction to human natural-killer NK-92 cell line or mouse T cell lymphoma EL4 cell line in the presence of Polybrene (8μg/mL final concentration) and spinoculated at 800 × g for 1.5 h. The cells were then incubated for 48 h before selection with puromycin at 1μg/mL.

## Lentiviral shRNAs for the knockdown of *LMP-1* in NKYS cell line

Lentiviral shRNA constructs expressing EGFP:T2A:Puro-scramble (Cat. #VB900168-2900uqb, VectorBuilder) and LMP-1-specific shRNAs were used . The sequences of the shRNAs are as follows: shCtrl: CAACAAGATGAAGAGCACCAA, sh*LMP-1*#1: AGCCACATTCATATTCTATTT, and sh*LMP-1*#2: CCTTTGCTCTCATGCTTATAA. Lentiviral particles were collected 48 h after co-transfection of HEK293FT cells with either scramble shCtrl or shRNAs targeting LMP-1, along with packaging vectors (pMDLg/pRRE, pRSV-Rev, and pCMV-VSV-G). For transduction, two million cells from NKYS cells were incubated with concentrated viral supernatant in the presence of 10 μg/mL polybrene (TR-1003, Sigma-Aldrich). The cell mixture was centrifuged at 1300 × g at 32°C for 90 min, followed by incubation at 37°C for an additional 12 h. The medium was then replaced with fresh complete medium. After 24 h, puromycin selection was initiated at 1 μg/mL to enrich successfully transduced cells. After four passages under selection, green fluorescent protein (GFP)-positive cells were further enriched using fluorescence-activated cell sorting (FACS). After sorting, puromycin was removed and the percentage of GFP-positive cells was periodically monitored to confirm the purity of the transduced cell population.

## *In vitro* irradiation of NKTCL cell lines

The NKYS cell line, transduced with either scrambled shCtrl or *LMP-1* shRNAs, and NK92 cells, transduced with either an empty vector (EV) or an LMP-1 overexpression (OE) vector, were seeded at a density of one million cells in four mL with the respective media per well, in triplicate. Cells were exposed to gamma radiation from a ^60^Co source irradiator (Gamma Chamber 4000A, Board of Radiation & Isotope Technology, India) at a dose rate of 0.42 Gy/min to doses up to 8 Gy. After 48 h of incubation, apoptosis was assessed using the APC Annexin V Apoptosis Detection Kit with PI (Cat. #640932, BioLegend) following the manufacturer’s protocol and an optimized setup on the FACSCelesta™ Cell Analyzer (BD Biosciences). Flow cytometry data were analyzed using FlowJo™ Software (v10.10.0, BD Biosciences), with gating strategies applied to accurately distinguish the apoptotic cell populations.

## *In vivo* irradiation of EL4 cell line in C57BL/6 mice

C57BL/6J mice (8 weeks old; The Jackson Laboratory) were housed under specific-pathogen-free conditions with ad libitum access to food and water, and acclimatized for ≥7 days prior to procedures. All experiments were approved by the Institutional Animal Care and Use Committee (IACUC protocol 2023/SHS/1818) and are reported in accordance with the ARRIVE 2.0 guidelines. Mice were anesthetized with isoflurane and injected subcutaneously with 5×10^5^ EL4 cells (pBABE empty vector or *LMP-1* overexpressing vector) in 100 µL of PBS using a 27-G needle. Mice were randomly assigned to one of four arms (*n* = 10 per arm; 6 males and 4 females per arm) in a 2×2 factorial design: EL4 cells transduced with pBABE EV or LMP-1–OE vector, each with or without local tumor irradiation at 4 Gy on days 8 and 15 post-implantation using a Gammacell 40 Exactor (¹³⁷Cs source) with image guidance and collimation. Mice were monitored for general health and tumor take every 2–3 days. Tumors were measured with calipers, and tumor volumes were calculated as (length × width²)/2. In accordance with the approved protocol, the humane endpoint was defined as a tumor volume ≥ 2,000 mm³ or a composite of welfare signs (e.g., ulceration), whichever occurred first; animals meeting this endpoint were euthanized and the event time recorded as the day of euthanasia. No post-endpoint measurements were analyzed (**Supplementary Table** **S9**).

## DNA fiber **assay**

Cells (5 × 10^5^ cells/well) were seeded in 6-well plates for 24 h. Cells were labelled for 15 min with 60 μM 5-chloro-2'-deoxyuridine (CldU) (Cat. #C6891-100 mg, Sigma Aldrich), followed by labelling with 250 μM 5-Iodo-2′-deoxyuridine (IdU) (Cat. #17125-5G, Sigma Aldrich) for another 15 min, before they were subjected to a 5 h treatment with hydroxyurea. Low IdU-/CIdU-tract length ratios indicate slower fork progression, whereas high ratios indicate faster fork progression after treatment. For the untreated condition, cells were harvested 7 min after labelling with IdU. Following treatment, labelled cells were harvested and resuspended in 1x PBS to a concentration of 2.5 × 10^5^ cells/mL, and the cell suspension was mixed with lysis buffer (200 mM Tris-HCl pH 7.4, 50 mM EDTA (Cat. #V4231, Promega), 0.5% SDS (Cat. #L3771-1KG Sigma Aldrich) on a glass slide. Slides were inclined at 45° to allow the suspension to spread on the slide. Upon drying, the DNA fiber spreads were fixed in a methanol-acetic acid solution at 4°C overnight. The following day, the DNA was denatured with 2.5 N HCl for 80 min. The slides were then blocked for 40 min in filtered blocking solution (2% BSA (Cat. #A7906, Sigma Aldrich), 0.1% Tween 20 (Cat. #H5151, Promega), 1x PBS; 0.22 µm (Cat. #10010023, Gibco)). Primary antibody incubation was subsequently performed using the anti-BrdU antibody (Cat. #BU1/75 (ICR1), Abcam) and mouse monoclonal anti-Bromodeoxyuridine antibody (Cat. #347580, Becton Dickinson,) in blocking buffer for 2.5 h at room temperature in the dark. Next, the secondary antibody was incubated with anti-mouse IgG Alexa 488 (Cat. A-11001, ThermoFisher) and anti-rat Cy3 (Cat. #712-166-1530, Immuno Research) in blocking buffer for 1 h at room temperature in the dark. Slides were mounted overnight using Prolong Antifade Gold (Cat. #P36930, Invitrogen). Images were acquired using an Axioimager Z1 (Zeiss) microscope, and at least 100 DNA fibers were quantified using ImageJ2 (v2.3.0 [22]).

## Western Blot

Experimental procedures for Western blotting were performed as described previously [3]. The antibodies used are listed in **Supplementary Table S10**.

## Statistical analysis

We used Fisher’s exact test [23] to identify associations between categorical variables, Wilcoxon’s rank-sum test [24] to identify differences in discrete and continuous variables, the Kaplan-Meier estimator [25] to estimate the overall and progression-free survival function from time-to-event data, the log-rank test [26] to compare survival between categorial features. *P*-values < 0.05 were deemed statistically significant.

## Clinical Characteristics of Patients with NKTCL

A total of 77 patients with extranodal nasal-type NKTCL formed the core cohort of this study and most of the tumors were collected at first diagnosis (72/77, 93.5%). This cohort predominantly consisted of males (61/77, 79.2%), which is consistent with known demographic of NKTCL [27]. The median age of the cohort at diagnosis was 46 years (range, 15 to 82 years). The majority of patients were of Chinese ethnicity (69/77, 89.6%), and cases from diverse backgrounds including Malays (*n* = 3), Indonesians (*n* = 3), Burmese (*n* = 1), and Emirati (*n* = 1) were also present in our study (**Supplementary Table S7**). For the various clinical features which we recorded for the cohort, 27.3% of the patients (21/77) were of advanced age (≥ 60 years old), 27.3% (21/77) were of late-stage (Ann Arbor Stage III – IV), 7.8% (6/77 ) were of high Eastern Cooperative Oncology performance status (ECOG PS ≥ 2, 1 missing), 44.7% (34/76, 1 missing) had elevated lactate dehydrogenase (LDH), 29.9% (23/77) had extranodal disease involving more than one anatomical site (E_site > 1), 49.3% (35/71, 6 missing) had detectable circulating pretreatment EBV. Among the clinical risk-factors, late-stage was most correlated with more than one extranodal site (rho = 0.38, *P* < 0.001), which was expected as disease dissemination would usually represent advanced disease. Furthermore, by using our previous developed genomic prognostic model for NKTCL, we were able to stratify 32.5% (25/77) patients into the high-risk genomic group 2. In summary, only 16.2% (12 of 74; 3 missing values) of patients had International Prognostic Index (IPI) risk-score of zero, which underscored the aggressive nature of NKTCL. Among the remaining patients, 48.6% (36/74) had a score of 1, 21.6% (16/74) had a score of 2, 12.2% (9/74) had a score of 3, and 1.4% (1/74) had a score of 4. Patients were generally treated with chemotherapy, radiotherapy or in combination and we collectively grouped 63.6% (49/77), 9.2% (7/76) and 81.6% (62/76) of them into radiotherapy, anthracycline-containing and asparaginase-containing regimens treatment-groups, respectively (**Supplementary Table S7**).

# Supplementary Figures

**Supplementary Figure S1**. **Mutational density of non-silent mutations across the viral *LMP-1* gene.** Histogram plot of 5-amino acid bins on non-silent mutations across the viral *LMP-1* gene. Counts of SNVs and small-indels were tabulated above and below the main y-axis with UniProt annotations as multi-colored horizontal bars at the bottom of the plot. Abbreviations: *LMP-1*, latent membrane protein 1; aa, amino acid; N, N-terminus; C, C-terminus; CTAR, C-terminus activating region; SNV, single-nucleotide variant.

**Supplementary Figure S2**. **IGV coverage tracks of samples with *RPMS1* deletions.** A snapshot of NKTCL samples’ depth-coverage track over the entire NC_007605 Epstein-Barr virus reference genome using IGV showing genomic-losses implicating the viral *RPMS1* gene (NC_007605: 138,302-160,581; in blue shade). Abbreviation: IGV, integrative genomics viewer; *RPMS1*, rightward reading frame in the BamHI-M and -S regions 1.

**Supplementary Figure S3**. **PCR-based Sanger sequences spanning across the breakpoints of viral copy-gains.** The PCR-based Sanger sequencing validations performed on the genomic gains of the viral *LMP-1* genes in samples, **(A)** NKTL6 and **(B)** NKTL7 are depicted here. Red arrows depicts where the copy-gain implicating *LMP-1* is across the EBV genome. The green and purple arrows on the top depth-coverage tracks depict where primer-pair were designed to amplify the genomic fragment flanking the breakpoint supporting the copy-gain of the *LMP-1* locus. The Sanger sequences were then pasted into NCBI Virus BLAST to yield their positional information on NCBI’s reference genomes. The corresponding zoomed-in screenshots of the Sanger read-alignment, from NCBI’s Virus BLAST, and Sanger-chromatogram are shown side-by-side for easy comparison on read-alignment and base-call qualities. Abbreviations: PCR, polymerase chain reaction; EBV, Epstein-Barr virus; *LMP-1*, latent membrane protein 1; NCBI, National Center for Biotechnology Information; EBV, Epstein-Barr virus; BLAST, Basic Local Alignment Search Tool

**Supplementary Figure S4**. **Long-read sequencing confirmed copy-gains of *LMP-1* within the viral genome of the NKYS cell line.** The depth-coverage track from WGS short-read sequencing, a long read spanning 39,933bp long across the EBV genome from Nanopore sequencing, chromatogram of Sanger sequence and the NCBI Virus BLAST results of the sanger sequence that confirmed the genomic copy-gain of *LMP-1* in the NKYS cell line. Abbreviations: WGS, whole-genome sequencing; NCBI, National Center for Biotechnology Information; EBV, Epstein-Barr virus; BLAST, Basic Local Alignment Search Tool

**Supplementary Figure S5**. **Short-read and long-read resequencing on the viral isolates of two NKTCL tumor samples confirmed viral genomic breakpoints of *LMP-1* copy-gains.** Cross-validation between WGS short-read, viral-isolate short-read and viral-isolate long-read resequencing data showing the rearrangements of EBV implicating the *LMP-1* locus for **(A)** NKTL6 and **(B)** NKTL7. Abbreviation: WGS, whole-genome sequencing.

**Supplementary Figure S6**. **Overall survival of patients with NKTCL by their copy-gain status of *LMP-1*.** The Kaplan-Meier curves for the OS (*n* = 76) of patients with NKTCL tumors, in our study cohort, harbouring *LMP-1^copy-gain^* and *LMP-1^wild-type^*. *P*-values were obtained by performing log-rank tests. Abbreviation: *LMP-1*, latent membrane protein 1; OS, overall survival; Gain, *LMP-1^copy-gain^* ; WT, wild-type.

**Supplementary Figure S7**. **Overall survival of patients with NKTCL who have received radiotherapy by their copy-gain status of *LMP-1*.** The Kaplan-Meier curves for the OS (*n* = 76) of patients with NKTCL tumors who have received radiotherapy in our study cohort, harbouring *LMP-1^copy-gain^* and *LMP-1^wild-type^*. *P*-values were obtained by performing log-rank tests. Abbreviations: OS, overall survival; NKTCL, natural-killer/T cell lymphoma; *LMP-1*, latent membrane protein 1; OS, overall survival; Gain, *LMP-1^copy-gain^* ; WT, wild-type.

**Supplementary Figure S8. Experimental design of *in vivo* mice work for studying the effect of irradiation and *LMP-1* expression. (A)** A schematic plot and **(B)** CONSORT diagram describing how the mice were grouped, treated and when they were measured to test the effect of irradiation on EV and LMP-1-OE EL4 cells. Abbreviations: *LMP-1*, latent membrane protein 1; IR, irradiation; EV, empty vector; OE, overexpressing.

**Supplementary Figure S9. Spaghetti plots of tumor volumes across different conditions to study the effect of irradiation and *LMP-1* on EL4 cells *in vivo*.** Spaghetti plots of individual tumor volumes from four groups of 10 (6 males and 4 females) C57BL/6 mice. C57BL/6 mice were subcutaneously implanted with either EV or LMP-1-OE EL4 cells. Mice were randomly assigned to one of four experimental groups: **(A)** EV (No IR), mice bearing EV tumors that received no irradiation; **(B)** EV (IR), mice bearing EV tumors treated with localized tumor irradiation; **(C)** OE (No IR), mice bearing LMP-1-OE tumors that received no irradiation; and **(D)** OE (IR), mice bearing LMP-1-OE tumors treated with localized tumor irradiation. Each spaghetti line represents an individual tumor volume. Tumor volumes were monitored longitudinally and displayed up to a maximum of 2,000 mm³ in accordance with the study endpoint. Fractional irradiations were given at 4 Gy on day 8 and 15 to the treatment-groups of remaining mice. Abbreviations: *LMP-1*, latent membrane protein 1; EV, empty vector; OE, overexpressing; IR, irradiation.

**Supplementary Figure S10. Relative changes of tumor-volumes in various conditions to study the effect of irradiation and *LMP-1 in vivo*.** Longitudinal EL4 xenografts response to irradiation plotted as relative tumor volume changes from baseline (Day 7). LMP-1-OE and EV were transfected into EL4 cells for the xenografts. Curves show mean ± SEM relative change from baseline (Day 7) for the four groups: EV (IR, red; no IR, blue) and OE (IR, green; no IR, orange). Fractional irradiation of 4Gy were given on day 8 and 15. Asterisks denote level of significance between the two treatment-groups, (EV vs OE) significance at each time-point: *P* < 0.05 (*), *P* < 0.01 (**), *P* < 0.001 (***). Abbreviations: *LMP-1*, latent membrane protein 1; EV, empty vector; OE, overexpression; IR, irradiation.

**Supplementary Figure S11. Baseline comparisons of LMP-1 in our overexpression and knockdown models.** Western-blots analysis of LMP-1 in our control EV and control cell lines as compared to their respective *LMP-1* overexpression and *LMP-1* knockdowns (sh*LMP-1*#1 and #2), NK92 and NKYS cell lines. Abbreviations: *LMP-1*, latent membrane protein 1; GAPDH, glyceraldehyde-3-phosphate dehydrogenase; EV, empty vector; sh. short hairpin; OE, overexpression; Ctrl, control; kDa, kilodalton.

**Supplementary Figure S12.** **Flow cytometric plots showing apoptotic levels in our *LMP-1* knockdown model from irradiation.** The flow cytometric plots for the concomitant PI (Comp-PI-A) and Annexin V (Comp-APC-A) staining of our established NK92 and NKYS stable cell lines after getting up to 8 Gy of irradiation. Bars show error bars with mean ± SEM with respect to baseline. Abbreviations: *LMP-1*, latent membrane protein 1; PI, Propidium Iodide; Gy, Gray; sh, short hairpin; Ctrl, control. Asterisks denote level of significance, *P* < 0.001 (***).

**Supplementary Figure S13. Knockdown of *LMP-1* lowered apoptotic activity from irradiation in NKYS cells.** Western blot analysis of γH2AX (post-8 h of irradiation for DNA damage) and cleaved caspase 3 (post-24 h of irradiation for apoptotic activity) in both scramble-control and two sets of *LMP-1*^knockdown^ NKYS cells, with and without exposure to 8 Gy of irradiation, respectively. Abbreviations: LMP-1, latent membrane protein 1; sh, short hairpin; IR, irradiation; Ctrl, control; kDa, kilodalton; γH2AX (Ser139), gamma H2A histone family member X (serine 139); β-actin, beta-actin.

**Supplementary Figure S14. Experimental design on using DNA fiber assay to assay replication fork stalls from *LMP-1* and DNA-damaging agent.** Schematic of single-molecule DNA fiber assay using nucleotide analogs (CldU and IdU), along with drug treatment, can evaluate an array of replication-forks parameters. Our stable cell lines were labelled with CIdU (0.25 h) and IdU (0.25 h). Subsequently, the cells were treated with HU for 5 h. Abbreviations: CldU, 5-chloro-2'-deoxyuridine; IdU, 5-iodo-2'-deoxyuridine; HU, hydroxyurea.

**Supplementary Figure S15. IGV coverage tracks of copy-wildtype samples across the *LMP-1* genic locus.** A snapshot of 4 NKTCL samples, without aberrant copy changes within their viral genomes, with depth-coverage track over the entire NC_007605 Epstein-Barr virus reference genome using IGV that was used as a “pool of normals” as a reference to call out viral copy-number variants. Abbreviation: natural-killer/T cell lymphoma, NKCL; *LMP-1*, latent membrane protein 1; IGV, integrative genomics viewer.

# References

1.	Chan JKC, Quintanilla-Martinez L, Ferry JA, Peh S-C. Extranodal NK/T-cell lymphoma, nasal type. In: Swerdlow SH, Campo E, Harris NL, Jaffe ES, Pileri SA, Stein H, et al., editors. WHO classification of tumours of haematopoietic and lymphoid tissues. 4th ed. Lyon: IARC Press; 2008. p. 285-8.

2.	Alaggio R, Amador C, Anagnostopoulos I, Attygalle AD, Araujo IBO, Berti E, et al. The 5th edition of the World Health Organization Classification of Haematolymphoid Tumours: Lymphoid Neoplasms. Leukemia. 2022;36(7):1720-48.

3.	Nairismagi ML, Tan J, Lim JQ, Nagarajan S, Ng CC, Rajasegaran V, et al. JAK-STAT and G-protein-coupled receptor signaling pathways are frequently altered in epitheliotropic intestinal T-cell lymphoma. Leukemia. 2016;30(6):1311-9.

4.	Chen S, Zhou Y, Chen Y, Gu J. fastp: an ultra-fast all-in-one FASTQ preprocessor. Bioinformatics. 2018;34(17):i884-i90.

5.	Li H. Aligning sequence reads, clone sequences and assembly contigs with BWA-MEM. arXiv:1303. 2013.

6.	Tarasov A, Vilella AJ, Cuppen E, Nijman IJ, Prins P. Sambamba: fast processing of NGS alignment formats. Bioinformatics. 2015;31(12):2032-4.

7.	Okonechnikov K, Conesa A, Garcia-Alcalde F. Qualimap 2: advanced multi-sample quality control for high-throughput sequencing data. Bioinformatics. 2016;32(2):292-4.

8.	Kim S, Scheffler K, Halpern AL, Bekritsky MA, Noh E, Kallberg M, et al. Strelka2: fast and accurate calling of germline and somatic variants. Nat Methods. 2018;15(8):591-4.

9.	Garrison E, Marth G. Haplotype-based variant detection from short-read sequencing. arXiv:1207.

10.	Chang X, Wang K. wANNOVAR: annotating genetic variants for personal genomes via the web. J Med Genet. 2012;49(7):433-6.

11.	Smigielski EM, Sirotkin K, Ward M, Sherry ST. dbSNP: a database of single nucleotide polymorphisms. Nucleic Acids Res. 2000;28(1):352-5.

12.	Tate JG, Bamford S, Jubb HC, Sondka Z, Beare DM, Bindal N, et al. COSMIC: the Catalogue Of Somatic Mutations In Cancer. Nucleic Acids Res. 2019;47(D1):D941-D7.

13.	Talevich E, Shain AH, Botton T, Bastian BC. CNVkit: Genome-Wide Copy Number Detection and Visualization from Targeted DNA Sequencing. PLOS Computational Biology. 2016;12(4):e1004873.

14.	Mermel CH, Schumacher SE, Hill B, Meyerson ML, Beroukhim R, Getz G. GISTIC2.0 facilitates sensitive and confident localization of the targets of focal somatic copy-number alteration in human cancers. Genome Biol. 2011;12(4):R41.

15.	Chen X, Schulz-Trieglaff O, Shaw R, Barnes B, Schlesinger F, Kallberg M, et al. Manta: rapid detection of structural variants and indels for germline and cancer sequencing applications. Bioinformatics. 2016;32(8):1220-2.

16.	Geoffroy V, Herenger Y, Kress A, Stoetzel C, Piton A, Dollfus H, et al. AnnotSV: an integrated tool for structural variations annotation. Bioinformatics. 2018;34(20):3572-4.

17.	Zhu H, Uuskula-Reimand L, Isaev K, Wadi L, Alizada A, Shuai S, et al. Candidate Cancer Driver Mutations in Distal Regulatory Elements and Long-Range Chromatin Interaction Networks. Mol Cell. 2020;77(6):1307-21 e10.

18.	O'Leary NA, Wright MW, Brister JR, Ciufo S, Haddad D, McVeigh R, et al. Reference sequence (RefSeq) database at NCBI: current status, taxonomic expansion, and functional annotation. Nucleic Acids Res. 2016;44(D1):D733-45.

19.	Quinlan AR, Hall IM. BEDTools: a flexible suite of utilities for comparing genomic features. Bioinformatics. 2010;26(6):841-2.

20.	Dobin A, Davis CA, Schlesinger F, Drenkow J, Zaleski C, Jha S, et al. STAR: ultrafast universal RNA-seq aligner. Bioinformatics. 2013;29(1):15-21.

21.	Li B, Dewey CN. RSEM: accurate transcript quantification from RNA-Seq data with or without a reference genome. BMC Bioinformatics. 2011;12:323.

22.	Rueden CT, Schindelin J, Hiner MC, DeZonia BE, Walter AE, Arena ET, et al. ImageJ2: ImageJ for the next generation of scientific image data. BMC Bioinformatics. 2017;18(1):529.

23.	Fisher RA. On the Interpretation of χ 2 from Contingency Tables, and the Calculation of P. Journal of the Royal Statistical Society. 1922;85(1).

24.	Mann HB, Whitney DR. On a Test of Whether one of Two Random Variables is Stochastically Larger than the Other. The Annals of Mathematical Statistics. 1947;18(1):50-60.

25.	Dinse GE, Lagakos SW. Nonparametric estimation of lifetime and disease onset distributions from incomplete observations. Biometrics. 1982;38(4):921-32.

26.	Mantel N. Evaluation of survival data and two new rank order statistics arising in its consideration. Cancer Chemother Rep. 1966;50(3):163-70.

27.	Kim TM, Lee SY, Jeon YK, Ryoo BY, Cho GJ, Hong YS, et al. Clinical heterogeneity of extranodal NK/T-cell lymphoma, nasal type: a national survey of the Korean Cancer Study Group. Ann Oncol. 2008;19(8):1477-84.
